# Impact of sex hormonal changes on tension-type headache and migraine: a cross-sectional population-based survey in 2,600 women

**DOI:** 10.1007/s10194-012-0475-0

**Published:** 2012-08-31

**Authors:** Necdet Karlı, Betül Baykan, Mustafa Ertaş, Mehmet Zarifoğlu, Aksel Siva, Sabahattin Saip, Güven Özkaya, Turkish Headache Prevalence Study Group

**Affiliations:** 1 Department of Neurology, School of Medicine, University of Uludağ, 16059 Bursa, Turkey; 2 Department of Neurology, Istanbul Faculty of Medicine, Istanbul University, Istanbul, Turkey; 3 Department of Neurology, Anadolu Health Center Hospital, Gebze, Kocaeli Turkey; 4 Department of Neurology, Cerrahpaşa School of Medicine, Istanbul University, Istanbul, Turkey; 5 Department of Biostatistics, School of Medicine, University of Uludağ, Bursa, Turkey

**Keywords:** Sex hormones, Headache, Migraine, Tension-type headache, Menstruation, Menopause

## Abstract

Sex hormones have some implications on headaches. The objective of the study was to investigate the effects of hormonal changes comparatively on tension-type headache (TTH) and migraine, in a population-based sample. A nationwide face-to-face prevalence study was conducted using a structured electronic questionnaire. 54.3 % of the migraineurs reported that the probability of experiencing headache during menstruation was high, whereas 3.9 % had headache only during menstruation. Forward logistic regression analysis revealed that menstruation was a significant trigger for migraine in comparison to TTH. On the other hand, nearly double the number of TTH sufferers reported “pure menstrual headache” compared to migraineurs (*p* = 0.02). Menstrual headaches caused significantly higher MIDAS grades. One-third of the definite migraineurs reported improvement during pregnancy and oral contraceptives significantly worsened migraine. Menopause had a slight improving effect on migraine compared to TTH. Sex hormonal changes have major impacts particularly on migraine; however, the effects of hormonal fluctuations on TTH should not be underestimated.

**Electronic supplementary material:**

The online version of this article (doi:10.1007/s10194-012-0475-0) contains supplementary material, which is available to authorized users.

## Introduction

The obvious difference in pain prevalence related to gender is well known, but the underlying mechanisms are not clearly identified. Female sex steroids may play an important role in this difference. The most important sex hormones, estrogen and progesterone, influence peripheral and central pain transmission via serotonergic, noradrenergic, glutamatergic, GABAergic and opioidergic neurotransmitter systems [1, 2].

Headache is among the most prevalent and disabling disorders and pain syndromes [3, 4]. Epidemiological data in different populations have clearly shown that headache was more prevalent in women compared to men [4]. This difference is best visible in migraine, and migraine prevalence is more than doubled in women [4]. Migraine prevalence does not differ between genders before adolescence. However, a significant prevalence increase occurs in females compared to males after menarche [5–8], and this point inevitably leads to the hypothesis that female sex hormones play a crucial role in migraine pathophysiology [2, 9]. Many studies investigating this intriguing relationship between migraine and hormonal changes in women, i.e., menstruation, menopause, pregnancy and use of oral contraceptives (OCs), have shown some support for this hypothesis [10–17]. As a result, in 2004, the International Headache Society (IHS) proposed the diagnostic criteria for pure menstrual and menstrual-related migraine without aura in the appendix of the second edition of the International Classification of Headache Disorders (ICHD-2) [18].

On the other hand, studies investigating the relationship between tension-type headache (TTH) and hormonal changes are limited. The results of these studies were contradictive: some indicated similar findings like migraine, while some others reported no significant associations between hormonal changes and TTH [10–14, 17, 19]. Interestingly Arjona et al. [20] suggested a new term “menstrual TTH”, a description which was only used for migraine beforehand. Although, there is some data suggesting a relationship between hormonal changes and TTH, no consensus has been reached so far about the role for sex hormones in TTH pathophysiology. This gap might probably prevent us from investigating the possible additional role of hormonal mechanisms in TTH pathophysiology and establishing a link between TTH and migraine in relation to female sex hormones [21].

In this face-to-face, cross-sectional, population-based study of 2,600 women, we aimed to investigate the effects of sex hormonal changes both on TTH and migraine comparatively for the first time in Turkey, a country located between Asia and Europe with a different cultural background.

## Methods

We designed a nationwide, community-based prevalence study in adults aged between 18 and 65 years, with face-to-face interviews by 33 specially trained general practitioner physicians using a structured electronic questionnaire. The involved general practitioners received a 2-day long training on headache before the study. The comprehensive interview form included all diagnostic questions based on the ICHD-2 criteria [18] for diagnoses of migraine and TTH within the last year. Definite TTH was diagnosed if the participants were not diagnosed with “definite” or “probable” migraine and fulfilled all TTH criteria. Women suffering from both migraine and TTH were classified as having migraine. By definition, the TTH group consisted only of pure TTH sufferers for our study purposes. ICHD-2 criteria were the case definition criteria for the diagnoses of definite and probable TTH and migraine. Ten dedicated questions about sex hormonal changes and relationship with headache were included in the questionnaire, which the female subjects were asked (Appendix A). The validated Turkish version of the Migraine Disability Assessment (MIDAS) Questionnaire was also applied to all relevant subjects with headache.

The study design has been described elsewhere in detail [22]. Briefly, a multi-stage sampling method was used. In the first stage, 21 cities were selected representing the characteristics of households in all seven geographical regions of Turkey based on the ratio of their population to the total population of Turkey in the year 2008, according to the Turkish Statistical Institute (http://report.tuik.gov.tr/reports/rwservlet?adnksdb2=&ENVID=adnksdb2Env&report=turkiye_yasgr.RDF&p_yil=2008&p_dil=1&desformat=html). In the second stage, the distributions of urban and rural populations, gender and age groups were all taken into account to choose the target population in these cities, to ensure that there would be no selection bias; 6,000 households were chosen with an acceptable error rate of ±1.3 %. With the guidance of the quotas for each city, the houses to be visited were determined using a simple random-sampling method in districts, streets and rural areas. Only one person was interviewed in each household to avoid any bias. A Kish sampling grid was used to select one person per household to be interviewed. A total of 6,000 households were visited. After excluding the households visited but not interviewed because of several reasons such as “refusing to be interviewed”, “having no time”, “non-presence at home”, etc., 89 % of the households had valid interviews. In the end, the statistical standard error was ±1.3 % within a confidence interval of 95 % for 5,323 interviews, as planned. The study was completed within 3 months in the year 2008.

The menstruation period was accepted as 3 days before and 5 days after the beginning of menstruation similar to some other studies [23–25]. We believe that this broader range for the time window would have provided a more reliable data acquisition in a retrospective study and there are many patients reporting this broader period for menstrual aggravation in our clinical experience. Furthermore, due to the lack of a definition of menstrual TTH, except in the Arjona et al.’s study [20], the same time window was also used for TTH.

We asked the subjects to evaluate the course of their headaches during menstruation, menopause, pregnancy and OC intake as follows: disappeared, decreased (decreased ≥50 % in frequency), no change, worsened; do not remember; do not want to answer. Subjects were instructed to evaluate the impact of hormonal changes and pregnancies on their headaches as an overall impact throughout their life and considering all pregnancies.

We tried to figure out a hormonal impact modeling of TTH and migraine by evaluating the impact of hormonal situations on headaches. Therefore, we evaluated the likelihood of (1) experiencing headache during menstruation, (2) improving effect of pregnancy and (3) menopause as well as (4) the worsening effect of OCs on headache and analyzed the answers in 11 possible statistical combinations (for example, risk of experiencing headache during menses + worsening effect of OCs or the former two + improving effect of pregnancy, etc.). The relationship between these variables was entered in the model and the dependent variables, which were migraine and TTH, were analyzed. We tried to predict the likelihood of diagnosis by this modeling.

SPSS 15 software statistical package (SPSS Inc., Chicago, IL, USA) was used for statistical analysis. Those patients who did not want to answer the questions were excluded from further analysis. Shapiro–Wilk test was used as the normality test. Continuous variables were compared using Mann–Whitney *U* test and Kruskal–Wallis test when the data were not normally distributed. The categorical variables were analyzed by Pearson Chi-square, Yates’ continuity correction and Fisher’s exact test, where appropriate. Among patients with migraine and TTH, a forward logistic regression analysis was employed to examine the independent sex hormonal predictors of headache as explained above. A *p* value <0.05 was considered as significant. The odds ratio was used as a measure of relative risk and the associated 95 % confidence intervals [CI] were also calculated.

## Results

A total of 5,323 participants (2,600 (48.84 %) women) were interviewed. The ages of participants ranged between 18 and 65 with a mean of 36.2 ± 12 years for women. The details of this previous epidemiological study have been published elsewhere [22].

A total of 640 women were diagnosed with definite migraine and 349 with probable migraine (989 total migraine; definite + probable), whereas 116 had definite TTH and 228 had probable TTH diagnosis (344 total; definite + probable).

### Menstruation and headache

Of the total migraineurs, 722 (73.0 %) reported that they still had menstrual cycles, whereas 115 individuals (11.63 %) did not want to answer. Of the total TTH sufferers, 233 (67.7 %) reported that they were still menstruating, whereas 64 subjects did not want to answer. The number of subjects still menstruating in both migraine and TTH groups was similar (*p* = 0.525). Subjects currently on OCs were included in the analyses.

The comparison between total migraine and total TTH groups (“yes” and “sometimes” were evaluated together) showed that the migraine group had an increased probability of experiencing a headache attack during menstruation (*p* < 0.001) (Table 1). The odds ratio of “highly likely” experiencing headache (in reference to no) in migraineurs was 3.12 (95 % CI: 2.14–4.54) when compared with TTH sufferers (see online supplementary files for further details).

**Table 1:** The probability of experiencing a headache attack during menstruation and similarity to non-menstrual headaches

Headache type	Most of the time *n* (%)	Sometimes *n* (%)	Never *n* (%)	Different *n* (%)	Similar *n* (%)
Definite migraine	252 (54.3 %)	90 (19.4 %)	122 (26.3 %)	158 (46.2 %)	184 (53.8 %)
Probable migraine	94 (36.4 %)	62 (24.0 %)	102 (39.5 %)	70 (44.9 %)	86 (55.1 %)
Total migraine	346 (47.9 %)^*^	152 (21.1 %)	224 (31.0 %)	228 (45.8 %)^**^	270 (54.2 %)
Definite TTH	23 (27.7 %)	29 (34.9 %)	31 (37.3 %)	11 (21.2 %)	41 (78.8 %)
Probable TTH	28 (18.7 %)	50 (33.3 %)	72 (48.0 %)	22 (28.2 %)	56 (71.8 %)
Total TTH	51 (21.9 %)	79 (33.9 %)	103 (44.2 %)	33 (25.4 %)	97 (74.6 %)

The comparisons were made between most of the time + sometimes against never (Pearson Chi-square, Yates’ continuity correction and Fisher’s exact tests were used where appropriate)

The menstrual period was defined as 3 days prior to and 5 days after the beginning of menstruation

Similarity of the menstrual headache was compared to the usual headaches outside the menstruation period as shown in the last two columns (%)

* *p* < 0.001; compared to total TTH

** *p* < 0.001; compared to total TTH

Forward logistic regression analysis revealed that the probability of experiencing headache during menstruation was significantly higher (*p* = 0.006, OR = 2.79 95 % CI: 1.33–5.85) in migraineurs.

When total migraineurs were compared with total TTH sufferers, migraineurs reported significantly more different headaches during menstruation than the usual ones (*p* < 0.001) (Table 1) (see online supplementary files for further details).

**Fig. 1:**
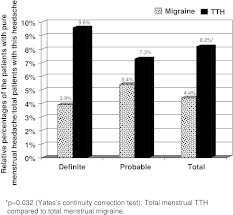
Pure menstrual headaches among migraine and tension-type headache patients

Only 32 (4.4 %) out of 722 menstruating migraineurs (total) and 19 (8.2 %) out of 233 menstruating TTH sufferers (total) reported pure menstrual headaches (Fig. 1) (*p* = 0.032).

### Pregnancy and headache

A total of 450 definite and 221 probable migraineurs reported that they had given birth to a child, whereas 78 definite and 120 probable TTH sufferers had given birth. The available information on the course of headache during pregnancy is shown in Table 2. Improvement and worsening of headaches were both significantly more frequent in the migraine group during pregnancy (Table 2) (see online supplementary files for further details). The OR for disappearance of headache (in reference to no change) in migraineurs during pregnancy was 2.56 (95 % CI: 1.53–4.29) in comparison to the TTH sufferers.

**Table 2:** The course of headache during pregnancy ('+' also contains those patients reporting decreased headaches after the 3rd month of pregnancy)

Headache type	Improved +	Worsened	No change
Definite migraine	162 (57 %)	31 (11 %)	91 (32)
Probable migraine	52 (46 %)	15 (13.3 %)	46 (40.7 %)
Total migraine	214 (53.9 %)^*^	46 (11.6 %)^**^	137 (34.5 %)
Definite TTH	12 (34.3 %)	1 (2.9 %)	22 (62.9 %)
Probable TTH	26 (42.6 %)	3 (4.9 %)	32 (52.5 %)
Total TTH	38 (39.6 %)	4 (4.2 %)	54 (56.3 %)^***^

Pearson Chi-square, Yates’ continuity correction and Fisher’s exact tests were used where appropriate

* *p* < 0.001, compared to total TTH

** *p* = 0.036 compared to total TTH

*** *p* < 0.001 compared to total migraine

### OC use and headache

A total of 117 definite and 58 probable migraineurs reported that they were currently on OCs or that they previously had used OCs. Both current and past OC users were included in the analyses. 16 definite and 34 probable TTH sufferers were either on OCs or had used OCs. The ratio of subjects reporting that they used OCs was similar between the headache groups and thus allowed healthy comparisons. The worsening effect of the OCs on migraine was significant when compared with TTH and the improvement rates were low, but similar between the groups. The details of the analyses are shown in Table 3 (see online supplementary files for further details).

**Table 3:** The course of headache during oral contraceptive use

Headache type	Disappeared	Decreased	Total improvement (disappeared + decreased)	Worsened	No change
Definite migraine (*n* = 117)	3 (4.0 %)	7 (9.5 %)	10 (13.5 %)	27 (36.5 %)	27 (36.5 %)
Probable migraine (*n* = 58)	3 (7.0 %)	2 (4.7 %)	5 (11.6 %)	12 (27.9 %)	21 (48.8 %)
Total migraine (*n* = 175)	6 (5.2 %)	9 (7.7 %)	15 (12.8 %)	39 (33.3 %)^*^	48 (41.0 %)
Definite TTH (*n* = 16)	1 (12.5 %)	0	1 (12.5 %)	0 (0 %)	6 (75.0 %)
Probable TTH (*n* = 34)	1 (5.0 %)	1 (5.0 %)	2 (10.0 %)	1 (5.0 %)	15 (75.0 %)
Total TTH (*n* = 50)	2 (7.1 %)	1 (3.6 %)	3 (10.7 %)	1 (3.6 %)	21 (75.0 %)^**^

Pearson Chi-square, Yates’ continuity correction and Fisher’s exact tests were used where appropriate

* *p* ≤ 0.001; Compared to total TTH

** *p* ≤ 0.001; Compared to total migraine

### Menopause and headache

**Fig. 2:**
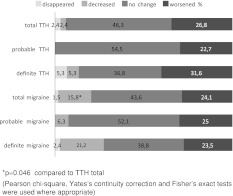
Course of headache during menopause

A total of 85 definite and 48 probable migraineurs and 19 definite and 22 probable TTH sufferers reported that they were in menopause. The distribution of the subjects in menopause did not show significant difference between the headache subgroups (*p* = 0.620). Menopause had a slight improving effect on migraine compared to TTH (*p* = 0.046) (Fig. 2) (see online supplementary files for further details).

### Sex hormonal impact modeling for headaches

The reported risk of experiencing headache during menstruation, impact of pregnancy, menopause and OCs on headaches and pure menstrual headache were all found to be significantly different in univariate analyses done between TTH and migraine groups as reported above, and a following forward logistic regression analysis showed that the only hormonal situation that was significantly different between the two headache types was the risk of experiencing headache during menses (*p* = 0.006, OR = 2.79 (95 % CI: 1.33–5.85), *B* = 1.028). All other significant differences in univariate analysis did not reach a significance level in multivariate analysis.

After analyzing these various effects of sex hormonal changes on headache, we tried to figure out whether we could come out with a hormonal impact modeling of migraine and TTH. In all combinations, those reporting high risk of experiencing headache during menses and worsening of headache during OC use were significantly higher in migraineurs compared to TTH sufferers (*p* = 0.003). The positive predictive value of this specific combination of hormonal changes for migraine is 28.9 %, which is very low. All other hormonal situations’ impact combinations were not significant.

### The effect of menstrual headaches on MIDAS

We further analyzed the relationship between the risk of experiencing headache during menstruation and MIDAS grade as an outcome. All primary headache sufferers (migraine and TTH), experiencing (highly likely or sometimes) headache during menstruation had significantly higher MIDAS grades (*p* < 0.001) (Mann–Whitney *U* test). The separate analysis revealed the same result for migraine (*p* < 0.001), but not for TTH (*p* = 0.711) (Table 4).

**Table 4:** The relationship between experiencing headache during menstruation and MIDAS grade

	Migraine presence of menstrual headache		TTH presence of menstrual headache		Migraine + TTH presence of menstrual headache	
	Yes	No	Yes	No	Yes	No
*n*	498	224	130	103	642	343
Median MIDAS grade	1^*^	1	1	1	1^**^	1
IQR	2	1	0	0	1	0

The interquartile range (IQR) is a measure of statistical dispersion in descriptive statistics, being equal to the difference between the upper and lower quartiles, IQR = Q3−Q1

* *p* < 0.001 (Mann–Whitney *U* test); compared to migraineurs without menstrual migraine headaches

** *p* < 0.001 (Mann–Whitney *U* test); compared to without menstrual headaches

## Discussion

We conducted a comparative investigation of sex hormonal milestones which might have an impact on both migraine and TTH, in the same population-based retrospective study. Our results revealed that sex hormonal changes affected migraineurs more frequently than TTH sufferers as expected [19, 26–29]. One of the novel and interesting results of this study was that significantly more TTH sufferers reported headaches limited to the menstrual period, even though more migraineurs in general reported headache aggravation during menstruation. Oral contraceptive pills had a significant worsening effect on migraine, whereas the impact of pregnancy was more prominent on migraine in both ways (improving and worsening). Menopause, on the other hand, had an improving effect on migraine.

### Menstruation and headache

Of all female migraineurs, 54.3 % reported that they were highly likely to experience headaches during menstruation. It was remarkable that migraine attacks during menstruation had a significant relationship with worsened overall MIDAS grade (*p* < 0.001). Some studies reported that migraine attacks during menstruation were more severe and longer than the usual attacks occurring at other times [30–32]. We speculate that this severity or altered perception of the migraine attack during menstruation might lead to worsened disability. Furthermore, our findings clearly indicate that headache caregivers should focus on the menstrual migraine attacks and treat them more vigorously. Better management of migraine attacks during menstruation might provide much more benefit on the outcome than expected.

Almost all studies reported that menstruation was a very strong trigger, both for migraine and TTH [8, 11, 12, 17, 20, 26]. Rasmussen et al. [10] indicated that significantly more TTH sufferers reported menstruation as a trigger for headache when compared with migraineurs, whereas some headache center-based studies contradicted this finding [19] or other population-based studies reported that menstruation appeared to be a risk factor in similar frequencies both for migraine and TTH [11]. This discrepancy is probably related to the methodological differences, the source of the study groups or the presence of mixed headache groups. In our study, definite and probable TTH groups included only pure TTH sufferers as the subjects were diagnosed with TTH after the subjects with migraine diagnosis were excluded. More than half of TTH sufferers as well as three-fourths of all migraineurs reported that they experienced headache during menses. Forward logistic regression analysis revealed that menstruation was a significantly higher trigger factor for migraine in comparison to TTH. We showed that menses was not a specific, but a very frequent triggering factor for female migraineurs in the general population. Furthermore, migraineurs reported that the headaches they experienced during menstruation had different characteristics than the usual ones (mostly increased severity during menstruation) (*p* < 0,001, compared to TTH). On the other hand, more than 25 % of TTH sufferers also reported different headaches during menses.

There is limited data on menstrual migraine (MM) prevalence. Couturier et al. [33] reported MM prevalence to be 3 % in the general population. In our study, MM prevalence was slightly higher (4.4 %). However, our menstrual period was defined to be longer (−3, +5 days of menstruation) than that they had reported (−2, +3). Another study by mail questionnaires included women aged 30–44 years in Norway, and pure MM and menstrually related migraine prevalences were 2.7 and 4.6 %, respectively [34]. Age differences, study design as interview versus questionnaire and climatic or sociocultural differences might have resulted in a lower prevalence rate of pure MM in Norway [34].

One of the most remarkable results of this study was the higher frequency of pure menstrual headache in TTH sufferers as shown in Fig. 1. Arjona et al. [20] proposed the term ‘menstrual TTH’ for the first time. Our results supported their proposition and pure menstrual TTH might actually be a prevalent and underestimated problem in the female population. As much as 8.15 % of all TTH sufferers experienced pure menstrual TTH in our population, which is still lower compared to 28.5 % in the study (which was outpatient clinic based) by Arjona et al. [20]. We speculate that the mental and physical stress caused by menses make the individual more prone to TTH. Further epidemiological and pathophysiological studies investigating other aspects and underlying mechanisms of this interesting relationship between the menstruation and TTH should be planned in the future.

### Pregnancy and headache

Almost one-third of the definite migraineurs reported improvement during pregnancy. Worsening effect of pregnancy on migraine seemed to be higher than that on TTH even if the logistic regression analysis did not support this. The great majority of TTH sufferers were not affected by pregnancy. It was already reported that headaches had a tendency to diminish during pregnancy, particularly in migraine, with a very wide improvement rate of 18–80 % in different studies [10, 12, 13, 15, 19, 35–38]. All these studies had different methodologies and different definitions of improvement, making them difficult to compare. The most similar methodology to ours was by Rasmussen et al. [10]. The improvement rates among migraineurs (31.9 vs. 48 %) and TTH sufferers (19.2 vs. 28 %) in our study were lower than that reported [10]. It should be noted, however, that more than 40 % of migraineurs and 50 % of TTH sufferers were unable to remember the course of headache during their pregnancies and this problem might have decreased the improvement rates in our study. More than 50 % of the remaining women reported improvement in migraine, which is comparable to the study by Rasmussen et al. [10]. Similar to the same study, significantly more migraineurs reported improvement during pregnancy compared to TTH (*p* = 0.003). On the other hand, the rate of worsening was also higher for migraine and lower for TTH (6.9 and 2.0 % respectively in our study) compared to that reported by Rasmussen et al. [10]. Another retrospective epidemiological study reported higher improvement rate compared to ours [38]. Due to our retrospective design, we could not investigate the differences in the course of migraine between the first and the following pregnancies.

### Oral contraceptives and headache

The worsening effect of OCs on migraine was reported more than a decade ago by Cupini et al. [15]. However, there is still a great discrepancy on the relationship of OC use and headache in the relevant literature [15, 37]. In some population-based studies, no difference was found regarding the prevalence of migraine or TTH related to the use of OCs [10–12], whereas another prospective population-based study showed that 3–5 % of women experienced worsening of migraine [39]. In a population-based study in Croatia, OCs were reported among the precipitant factors of both migraine and TTH with similar rates [11]. Machado et al. reported slightly higher worsening ratio in migraineurs [40]. Our results showed a prominent worsening effect of OCs on migraine compared to TTH among the present and previous users similar to the Head-Hunt study [35]. The differences in the literature might reflect different methodologies of the studies or different subgroups of migraineurs, but there could also be some different sociocultural perceptions about the use of OCs in different populations.

### Menopause and headache

Migraine sufferers reported a significant decrease in headache frequency during menopause in comparison to TTH sufferers. Only 17.3 % of the migraineurs reported improvement during menopause, but the number of evaluated subjects with migraine in menopause was somewhat low (*n* = 113) in our study because of the subjects who could not recall the real effect. Neri et al. reported an improvement in almost 2/3 of migraineurs, whereas headache worsened or did not change in 70 % of TTH subjects [14]. Likewise, in our study worsened or unchanged headaches were about 73 % in our TTH subjects; but the improvement rate of migraine was significantly lower compared to this study [14]. Furthermore, in another population-based study, no significant association was reported between migraine and menopause in women aged between 40 and 74 years [41]. As the population-based data concerning the relation between TTH and menopause was very limited, it was difficult to compare our data with other studies.

There are several possible limitations of this study. First of all, recall bias was the major limitation, which could be expected in population-based retrospective studies. We diagnosed the subjects with migraine and TTH during the last year, whereas the questioned hormonal changes could happen at any time or even many years before. We do not exactly know if at that time the questioned subjects’ headache diagnoses were identical to those recorded during the last year. Therefore, the results cannot be generalized to the whole life period. The results should be evaluated with caution only for a certain lifetime period, but not for whole lifetime. Furthermore, our evaluation of the course of subjects’ past headaches during different hormonal situations without objectively measuring hormonal levels might have led to decreased objectivity. However, to our knowledge this is the first population-based epidemiological study investigating the impact of major hormonal fluctuations in women, both for migraine and also for TTH comparatively. To date, only some of the sex hormonal situations were investigated in population-based studies and most of them were limited to migraine. Besides, those studies investigating sex hormonal fluctuations are mostly from North America and West Europe. Thus, there is a definite need for epidemiological studies investigating these issues in different populations, as the influence on TTH and migraine across the world could vary from one country to another. This first study investigating the relationship between sex hormones and headache in Turkish women came from a different part of the world, which reflected different sociocultural and demographic characteristics. Face-to-face interviews and trained general practitioner physicians performing these interviews increased the power of our study in comparison to mailed questionnaires. The participation rate (89 %) was very satisfactory for such a large epidemiologic study and higher than many studies [10, 11, 16].

In conclusion; hormonal fluctuations appeared to have an impact, at least to some degree, on migraine as well as on TTH. Hormonal effects on TTH should not be underestimated, and there are a considerable number of women having pure menstrual TTH. More experimental and clinical studies are needed to evaluate the real effect of sex hormones on TTH.

### Electronic supplementary material

Below is the link to the electronic supplementary material.

Supplementary material 1 (DOC 283 kb)

## Appendix 1

Questions about hormonal situations

## Appendix 2: Turkish Headache Prevalence Study Group (alphabetical order)

Betül Baykan M.D.

Mustafa Ertaş M.D.

Necdet Karlı M.D.

Elif Kocasoy Orhan M.D.

Ayşe Emel Önal M.D.

Sabahattin Saip M.D.

Aksel Siva M.D.

Mehmet Zarifoğlu M.D.
