# Supplementary material for: Impact of sex hormonal changes on tension-type headache and migraine: a cross-sectional population-based survey in 2,600 women
Source: J Headache Pain. 2012 Aug 31;13(7):557–65. doi: 10.1007/s10194-012-0475-0 (PMC3444543; doi:10.1007/s10194-012-0475-0)
Supplement: Supplementary file 1 — Supplementary material 1 (DOC 283 kb) [file 10194_2012_475_MOESM1_ESM.doc]

IMPACT OF SEX HORMONAL CHANGES ON THE TENSION-TYPE HEADACHE AND MIGRAINE: A CROSS-SECTIONAL POPULATION BASED SURVEY IN 2600 WOMEN

(THE JOURNAL OF HEADACHE AND PAIN)

Necdet Karlı^1^ M.D. Prof; Betul Baykan^2^, M.D., Prof.; Mustafa Ertaş^3^, M.D., Prof; Mehmet Zarifoğlu^1^ M.D., Prof; Aksel Siva^4^ M.D, Prof; Sabahattin Saip^4^ M.D., Prof; Güven Özkaya^5^ PhD and Turkish Headache Prevalence Study Group

^1^University of Uludağ, School of Medicine, Department of Neurology

^2^ Istanbul University, Istanbul Faculty of Medicine, Department of Neurology

^3^ Anadolu Health Center Hospital, Department of Neurology

^4^ Istanbul University, Cerrahpaşa School of Medicine, Department of Neurology

^5^ University of Uludağ, School of Medicine, Department of Biostatistics

***Corresponding Author:***

Necdet Karlı

Address: University of Uludağ, School of Medicine, Department of Neurology 16059 BURSA / TURKEY

E-MAIL: [nkarli@yahoo.com](mailto:nkarli@yahoo.com), [nkarli@uludag.edu.tr](mailto:nkarli@uludag.edu.tr)

TEL:+90-533 3644086

Fax: +90-224 4429177

**RESULTS**

*Menstruation and Headache*

Of the 342 definite migraineurs reporting that they “highly likely” or “sometimes” experienced migraine attacks during menstruation, 158 (49.2%) subjects were sure that their menstrual migraine attacks were different than the usual ones. When total migraineurs were compared with total TTH sufferers, migraineurs reported significantly more different headaches during menstruation than the usual ones (p‹0.001) (Table 1).

Only 18 (3.9%) out of 433 migraineurs reported pure menstrual headaches. It is remarkable that significantly more TTH (total) sufferers reported pure menstrual headache compared to migraine (total) sufferers (Fig 1) (p=0.032).

Table 1: The probability of experiencing a headache attack during menstruation and similarity to non-menstrual headaches

| Headache Type | Most of the time  n (%) | Sometimes  n (%) | Never  n (%) | Different characteristics  n (%) | Similar Headache  n (%) |
| --- | --- | --- | --- | --- | --- |
| Definite Migraine | 252(54.3%)*,**,*** | 90 (19.4%) | 122 (26.3%) | 158 (46.2%) ^a,b^ | 184  (53.8%) |
| Probable Migraine | 94 (36.4%) | 62 (24.0%) | 102 (39.5%) | 70 (44.9%) ^c,d^ | 86 (55.1%) |
| Total Migraine | 346 (47.9%)^†^ | 152 (21.1%) | 224 (31.0%) | 228 (45.8%)^e^ | 270 (54.2%) |
| Definite TTH | 23 (27.7%) | 29 (34.9%) | 31 (37.3%) | 11 (21.2%) | 41 (78.8%) |
| Probable TTH | 28 (18.7%) | 50 (33.3%) | 72 (48.0%) | 22 (28.2%) | 56 (71.8%) |
| Total TTH | 51 (21.9%) | 79 (33.9%) | 103 (44.2%) | 33 (25.4%) | 97 (74.6%) |

*p‹0,001 (compared to probable migraine), **p=0,038 (compared to definite TTH), ***p‹0,001 (compared to probable TTH),

^†^ p‹0,001 (compared to TTH)

^a^ p‹0,001 (compared to definite TTH), ^b^p=0,004 (compared to probable TTH), ^c^p=0,002 (compared to definite TTH), ^d^p=0,014 (compared to probable TTH); ^e^ p‹0,001 (compared to TTH).

Please note that the comparisons were made between most of the time+sometimes against never. (Pearson chi-square, Yates’s continuity correction and Fisher’s exact tests were used where appropriate)

Also note that the menstrual period was defined as 3 days prior to and 5 days after the beginning of the menstruation.

Similarity of the menstrual headache was compared to the usual headaches outside the menstruation as shown in gray area (%).

Table 2: The Course of Headache during Pregnancy (+ also contains those patients reporting decreased headaches after the 3rd month of pregnancy)

| Headache Type | Improved + | Worsened | No Change |
| --- | --- | --- | --- |
| Definite Migraine | 162 (57%)*, ** | 31 (11%) | 91 (32%)^†, ††^ |
| Probable Migraine | 52 (46%)*** | 15 (13.3%) | 46 (40.7%)^†††^ |
| Total Migraine | 214 (53.9%)^****^ | 46 (11.6%)^a^ | 137 (34.5%) |
| Definite TTH | 12 (34.3%) | 1 (2.9%) | 22 (62.9%) |
| Probable TTH | 26 (42.6%) | 3 (4.9%) | 32 (52.5%) |
| Total TTH | 38 (39.6%) | 4 (4.2%) | 54 (56.3%)^††††^ |

*p=0.001, Compared to definite TTH; ** p=0.008, Compared to probable TTH; *** p=0,041, Compared to definite TTH; **** p<0.001, Compared to total TTH;

^†^p‹0,001, Compared to definite TTH; ^††^p=0.004, Compared to probable TTH; ^†††^p=0.035, Compared to definite TTH; ^††††^ p‹0,001, Compared to total migraine;

^a^p=0.036 Compared to total TTH (Pearson chi-square, Yates’s continuity correction and Fisher’s exact tests were used where appropriate)

Table 3: The Course of Headache during Oral Contraceptive Use

| Headache Type | Disappeared | Decreased | Total Improved  (Disappeared+decreased) | Worsened | No Change |
| --- | --- | --- | --- | --- | --- |
| Definite Migraine  (n=117) | 3 (4.0%) | 7 (9.5%) | 10 (13.5%) | 27 (36.5%)^†,††^ | 27 (36.5%) |
| Probable Migraine  (n=58) | 3 (7.0%) | 2 (4.7%) | 5 (11.6%) | 12 (27.9%)^†††^ | 21 (48.8%) |
| Total Migraine  (n=175) | 6 (5.2%) | 9 (7.7%) | 15 (12.8%) | 39 (33.3%)^††††^ | 48 (41.0%) |
| Definite TTH  (n=16) | 1 (12.5%) | 0 | 1 (12.5%) | 0 (0%) | 6 (75.0%)^*^ |
| Probable TTH  (n=34) | 1 (5.0%) | 1 (5.0%) | 2 (10.0%) | 1 (5.0%) | 15 (75.0%)^**,***^ |
| Total TTH  (n=50) | 2 (7.1%) | 1 (3.6%) | 3 (10.7%) | 1 (3.6%) | 21 (75.0%)^****^ |

*p=0.044 (Compared to definite migraine); **p=0.002 (Compared to probable migraine); ***p=0.04 (Compared to probable migraine); ^****^p≤0.001 (Compared to total migraine);

^†^ p=0.039 (compared to definite TTH); ^††^p=0.003 (Compared to probable TTH); ^†††^p=0.042 (Compared to probable TTH); ^††††^p≤0.001 (Compared to total TTH)

(Pearson chi-square, Yates’s continuity correction and Fisher’s exact tests were used where appropriate)

Figure 2: Course of headache during menopause

^*^P=0.046 (Compared to probable migraine); ^**^p=0.019 (Compared to probable TTH); ^***^p=0.046 (Compared to TTH total) (Pearson chi-square, Yates’s continuity correction and Fisher’s exact tests were used where appropriate)
